# Refractory Paraneoplastic Diarrhea Secondary to Adenocarcinoma of the Lung: A Case Report and Literature Review

**DOI:** 10.1155/2020/8825845

**Published:** 2020-07-25

**Authors:** Lauren Howdershelt, Michael J. Forte, Rahul G. Sangani

**Affiliations:** ^1^Department of Family Medicine, West Virginia University, 1 Medical Center Dr., Morgantown WV 26506, USA; ^2^Department of Medicine, Section of Pulmonary, Critical Care and Sleep Medicine, West Virginia University, 1 Medical Center Dr., Morgantown WV 26506, USA

## Abstract

Paraneoplastic diarrhea is a commonly described complication of gastrointestinal tract or endocrine malignancies. It is an extremely rare complication of lung adenocarcinoma, with only one previously reported case in the literature. A 46-year-old female with newly diagnosed stage IVb lung adenocarcinoma presented to our intensive care unit in hypovolemic shock with symptoms suggestive of diabetes insipidus (DI) as well as profuse large volume watery diarrhea. Exhaustive serological and microbiological workup revealed the diarrhea to be paraneoplasitc in nature. This case represents the second known case of paraneoplastic diarrhea secondary to lung adenocarcinoma. Clinicians should be aware of this rare phenomenon.

## 1. Introduction

Paraneoplastic diarrhea has been seen in tumors which primarily originate in the gastrointestinal tract or endocrine system. Lung cancer, specifically lung adenocarcinoma, can develop other paraneoplastic syndromes of hypertrophic osteoarthropathy and trousseau syndrome. Only one previous case of paraneoplastic diarrhea in the setting of lung adenocarcinoma has been described. The previous report described five months of secretory diarrhea in a patient with known adenocarcinoma of the right upper lung. The patient underwent a full workup showing an elevated chromogranin-A [1]. The patient's diarrhea resolved after surgical resection. We present a second case of paraneoplastic diarrhea in the setting of lung adenocarcinoma.

## 2. Case Report

A 46-year-old white female with recently diagnosed, untreated stage IVb adenocarcinoma of the right lung, as well as history of bipolar disorder, previously on lithium, presented to the hospital with abdominal pain, nausea, nonbloody nonbilious vomiting, diarrhea, fevers, and chills. Workup at the outside facility showed progression of disease with new pleural and hepatic metastasis. She was treated empirically for sepsis with antimicrobials and antifungals due to suspected infected MediPort, which was removed. Microbiological workup was unremarkable. She was transferred to our facility due to ongoing circulatory shock of unknown etiology.

On arrival to our hospital, she was persistently hypotensive despite multiple fluid boluses and was noted to have a high urine output (maximum of 13 L in 24 hours) concerning for diabetes insipidus (DI) along with profuse, high output of watery diarrhea. She was diagnosed with DI given her urine osmolarity of 202 mOsm/kg, urine specific gravity of 1.005, and urine sodium of 47 mOsm/kg in conjunction with a serum osmolarity of 298 mOsm/kg. Based on nephrology recommendations, she was continually treated with desmopressin, hydrochlorothiazide (HCTZ), and triamterene for mixed central and nephrogenic DI process.

**Figure 1:**
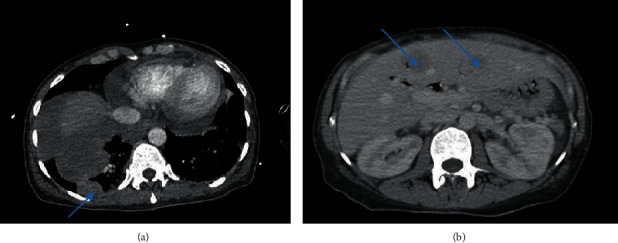
(a) New pleural based metastasis (blue arrow) with known large right lung mass and (a) 2 new metastatic liver lesions (blue arrows).

Despite her urine output decreasing, her stool output remained high, averaging 2700 mL per day. Clostridium difficile toxin and stool cultures were negative. New pleural and liver metastasis were visible on a repeat CT abdomen and pelvis as part of the workup with no other abnormalities seen in the GI tract (Figures 1(a) and 1(b)). Despite trials of loperamide, stool bulking agents, and later diphenoxylate/atropine, her diarrhea persisted. In addition, our gastroenterology colleagues deemed her too unstable for any endoscopic evaluation including colonoscopy. Given her history, the possibility of a paraneoplastic phenomenon was explored. A complete serological workup revealed only significantly elevated levels of chromogranin A and calcitonin (Table 1). Her overall conditioned continued to deteriorate and she developed multiorgan failure, refractory pain symptoms, and continued high stool output. Given her overall illness, she was deemed not a candidate for treatment of malignancy and opted for hospice care.

## 3. Discussion

Given the significant elevation in Chromogranin A and calcitonin in the setting of otherwise normal hormonal markers and negative infectious workup, we believe that the profuse, watery diarrhea experienced by our patient was a paraneoplastic phenomenon incited by her lung adenocarcinoma.

Typically, lung adenocarcinoma is associated with hypertrophic osteoarthropathy and not diarrhea. Of the three main types of lung cancer, small cell lung cancer has the highest rate of developing a paraneoplastic syndrome via the release of an endogenous substance and at times may result in free water retention or GI dysmotility [1]. However, secretory diarrhea defined as diarrhea that generates excessive amounts of stool with no osmotic gap and persists with fasting, secondary to a neoplasm is rarely reported in the literature [2].

Secretory diarrheas are more commonly associated with pancreatic neuroendocrine tumors such as gastrinomas, vasoactive intestinal peptide- (VIP-) omas, somatostatinomas, and carcinoid tumors. Our patient had normal or undetectable levels of these three pancreatic hormones essentially ruling these out. We additionally ruled out carcinoid tumor via normal levels of serotonin and urine metanephrines and the liver metastasis was known to be of lung origin.

With similar manifestations of diarrhea and cutaneous flushing, medullary thyroid cancer originates in the parafollicular C cells of the thyroid that produce calcitonin. This paraneoplastic phenomenon is often found in advanced medullary thyroid carcinoma and is due to elevated systemic calcitonin [3]. Calcitonin levels of >10 pg/mL often manifests in secretory diarrhea [4]. Our patient was found to have a calcitonin level of 229 pg/mL; however, no tumors were detected within her thyroid on imaging and had normal thyroid function panel.

Even though lung cancer has the largest association with paraneoplastic syndromes secretory diarrhea has rarely been found and few case reports exist within the literature. Tischer et al. [5] proposed secretory diarrhea as an atypical paraneoplastic syndrome in a patient with chronic diarrhea for three months who found relief from diarrhea after the resection of a solitary pulmonary nodule found to be small cell lung cancer. The endogenous molecule was not discovered. Later, adrenocorticotropic hormone (ACTH) and calcitonin causing diarrhea and metabolic abnormalities was found in the setting of small cell lung cancer [6]. Diarrhea associated with large cell carcinoma has been described in the literature twice; once in the setting of elevated VIP and calcitonin [7] and another in the case of elevated gastrin levels [8]. A similar scenario of paraneoplastic diarrhea in the setting of adenocarcinoma with elevated chromogranin-A to our case has been described. The authors reported that profuse diarrhea, once requiring up to doses of 12 mg of loperamide daily to control, completely resolved following surgical resection of the tumor [1].

In our case, the disease was too far advanced to consider surgical treatment; however, she demonstrated elevated chromogranin-A similar to the previous report with adenocarcinoma. Thus, we postulate that our patient's secretory diarrhea was due to the elevated chromogranin-A and calcitonin. We also hypothesize that the patient might have exhibited concomitant neuroendocrine features on her predominant histopathology of adenocarcinoma, but unfortunately, her pathology slides were not able to be transferred to our facility for further assessment. This case emphasizes the need for increased awareness among the clinicians regarding uncommon phenomenon of paraneoplastic diarrhea leading to critical illness. Early surgical management likely is key for resolution, as medical management alone carries a high risk of failure.

## Figures and Tables

**Table 1:** 

Patient's stool studies	Normal values in parenthesis
*Infectious disease*	
Stool leukocytes	No PMNs
Stool culture	Vibrio-negative
	Salmonella-negative
	Campylobacter-negative
	Shigella-negative
Clostridium difficile toxin PCR assay	Negative
*Parasitic studies*	
Ova and parasites screen	Negative
Cryptosporidium/Giardia	Negative
*Endocrine studies*	
5-HIAA, 24-hour urine	4.7 (<8 mg/24 hours)
ACTH	18.8 (6.0-59 pg/mL)
Cortisol (p.m.)	9.3 (2.0-14.0 ug/dL)
Calcitonin	229 (<7.6 pg/mL)
Chromogranin-A	152 (<92 ng/mL)
Gastrin	52 (0-180 pg/mL)
Serotonin	131 (<230 ng/mL)
Somatostatin	9 (up to 25 pg/mL)
VIP, plasma	Undetectable

Key: HIAA = 5-hydroxyindoleacetic acid, VIP = vasoactive intestinal peptide.
